# Editorial: Chronic autoimmune arthritis, infections and vaccines

**DOI:** 10.3389/fimmu.2022.1058152

**Published:** 2022-10-21

**Authors:** A. Picchianti Diamanti, M. M. Luchetti, E. Nicastri, M. M. Rosado, B. Laganà

**Affiliations:** ^1^ Department of Clinical and Molecular Medicine, “Sapienza” University, S. Andrea University Hospital, Rome, Italy; ^2^ Department of Clinical and Molecular Sciences, Marche Polytechnic University, Ancona, Italy; ^3^ Clinical Division of Infectious Diseases, National Institute for Infectious Diseases Lazzaro Spallanzani-Istituti di Ricovero e Cura a Carattere Scientifico (IRCCS), Rome, Italy; ^4^ Department of Internal Clinical Sciences, Anesthesiology and Cardiovascular Sciences, Sapienza University of Rome, Rome, Italy

**Keywords:** SARS-CoV-2 infection, COVID19, autoimmune disease, arthritis, vaccines, immunosuppressive therapy

The link between autoimmunity and infection continues to represent an intriguing immunologic conundrum for scientist and a frequent clinical challenge for patients and physicians.

Patients with chronic autoimmune arthritis indeed have an increased risk of infections, mainly due to the dysregulation of their immune system and the use of immunosuppressive therapy. Infections in these patients are more frequent, have a more severe clinical course, eventually with prolonged viral persistence, compared to the general population and represent a frequent cause of death.

Besides, infections can trigger autoimmune diseases *via* different immunologic mechanisms such as molecular mimicry, epitope spreading, by-stander activation and can also induce disease relapses.

SARS-CoV-2 infection represents a dramatic example of this complex connection.

It is known, indeed, that different autoimmune manifestations can complicate SARS-CoV-2 infection such as uncontrolled host-immune response leading to life-threatening condition known as cytokine release syndrome, or autoimmune hemolytic anemia, immune thrombocytopenic purpura, Guillain-Barre syndrome, and the detection of different autoantibodies.

This Research Topic includes seventeen contributions, fifteen original articles and two review articles, providing several new insights into the efficacy and safety of SARS-CoV-2 vaccine in autoimmune patients, immunologic biomarkers for diagnosis and therapeutic outcome of autoimmune arthritis.

The work of D’Abramo et al underlines the need of a prompt, combined multi-target tailored therapy for immunosuppressed patients with severe COVID-19. They described a case series of 21 COVID-19 patients under B cell depletion therapy, effectively treated with a combined therapy based on intravenous remdesevir and steroid associated with SARS-CoV-2 monoclonal antibodies against Spike glycoprotein and/or hyper-immune convalescent plasma

Sjowall et al compared SARS-CoV-2 antibodies in longitudinal samples collected prior to vaccination with Systemic Lupus Erythematosus (SLE) progression and antinuclear antibody levels. Their data from early 2021 indicate that a large proportion of Swedish SLE patients had serological signs of exposure to SARS-CoV-2 but apparently with a minor impact on the SLE course.

The development of several SARS-CoV-2 vaccines represented a major step-forward, but it raises special concerns for patients with autoimmune arthritis such as the choice of the best vaccine formulation and the management of ongoing immunosuppressive treatment in infected patients or during the scheduled vaccination.

Picchianti Diamanti et al provided an updated review of the main controversial issues in terms of safety, efficacy and effectiveness of SARS-CoV-2 vaccines in autoimmune patients and the role of immunosuppressive therapy. Immunomodulating agents such as dexamethasone and other biological agents (ie, tocilizumab, sarilumab, anakinra and baricitinib) demonstrated clinical and virological efficacy for the management of COVID-19 patients, however, they have to be tailored to COVID-19 severity and clinical setting, considering the strict correct window of opportunity to optimize patient’s outcome. On the other hand, autoimmune patients are a priority group for vaccination, but special measures should be adopted to improve vaccine safety and effectiveness, such as temporary suspension of some immunosuppressive drugs and the preferential use of mRNA-based vaccine

In their research article Picchianti Diamanti et al, showed for the first time that antibody-specific and whole-blood spike-specific T-cell responses induced by the COVID-19 mRNA-vaccine are present in the majority of Rheumatoid Arthritis (RA) patients, who underwent a strategy of temporary suspension of immunosuppressive treatment during vaccine administration, in the absence of disease relapses and major adverse events. However, the levels of specific responses were lower than Health Care Workers (HCWs) and reduced by some ongoing immunosuppressive therapy, in particular by the CTLA-4Ig abatacept.

In a following work on the same patient population Farroni et al, deeply characterized the kinetic of both humoral and cellular immune responses to BNT162b2 vaccine. The authors showed a significant reduction of the humoral response after 6 months from the first dose in both HCWs and RA patients regardless of the immunosuppressive therapy, whereas the T-cell response remained mostly stable. Looking at the T and B cell response overall, they stratified patients in full responders (both humoral and cellular, 40%), partially responders (only humoral or only T-cell response, 51%) and not responders (9%). The last two groups are likely to be at higher risk for COVID-19 despite the complete vaccination, underlining the need for a tailored strategy such as longer suspension of some immunosuppressive drugs and earlier adoption of a booster dose.

Through millions of people being vaccinated with COVID-19 mRNA vaccine is not surprising that rare reports of adverse events are emerging. In their Research article, Luchetti et al, suggests that the anti-Spike antibodies may play a key role in the induction of an abnormal and deregulated immune response. They evaluated the onset of clinical and laboratory immune manifestations related to COVID-19 or SARS-CoV-2 vaccination in a large cohort of hospitalized patients, with recently SARS-CoV-2 infection, or with autoimmune rheumatic diseases (ARDs) in remission and flared after SARS-CoV-2 infection, or finally outpatients with symptoms of probable immune-mediated origin after SARS-CoV-2 vaccination. They observed different clinical manifestations of ARD such as arthralgia/myalgia, pericarditis, thrombocytopenia as well as some cases of newly diagnosed ARD after the recovery from COVID-19 as well as after SARS-CoV-2 vaccination.

In the clinical case described by Hakroush and Tampe, there was a temporal association between the second dose of BNT162b2 vaccination and the development of anti-neutrophil cytoplasmic antibody associated vasculitis (AAV), rhabdomyolysis and pauci-immune crescentic glomerulonephritis (GN) suggesting a strong/uncontrolled neutrophilic immune response to mRNA vaccine as a potential trigger. Prompted treatment with intravenous cyclophosphamide followed by oral prednisone rescued kidney function, proteinuria dropped down and serological testing revealed that also ANCA IF turned negative.

Watanabe et al described a case of new-onset RA following COVID-19 vaccination. Flares or new-onset of autoimmune disorders have been reported soon after the COVID-19 vaccination, however in such a case, serum cytokine levels, after vaccination, resembled a typical genuine RA. Indeed, interleukin-6, tumor necrosis factor-alpha, type I interferon, were elevated at the active phase, whereas remission induced by methotrexate and tocilizumab was accompanied by a marked reduction of these cytokines.

Different interesting manuscripts focused on the pathogenesis, early diagnosis and therapy of RA were also submitted.

An important role in the pathogenesis of RA is played by the family of peptidylarginine deiminases (PADs) that have been linked to the anticitrullinated protein antibodies (ACPA) production. In their case-control study, Guzman-Guzman et al. found that the TTT haplotype of SNPs on the PADI2 gene confers genetic susceptibility to RA and radiographic joint damage in women from southern Mexico.

The experimental study of Brevet et al, gives a very interesting insight into the different roles that autoantibodies could have in RA. In fact, anti-carbamylated fibrinogen IgG antibodies (ACa-Fib IgG) were associated with a more inflammatory and erosive disease at baseline, and maybe correlated with systemic inflammation, but not with rapid radiological progression, which remains strongly related to ACPA antibodies.

Microparticles, also called extracellular vesicles (EVs), are small membrane-coated vesicles that are released from various cells during cell activation and apoptosis thus can be a source of autoantigens. They can also have important procoagulant properties based on the availability of phosphatidylserine (PS) exposed on the surface after stimulation. Stojanovic et al, found elevated levels of circulating EVs in patients with established RA, in relation to the inflammatory burden and coagulation activation in the disease.

Early diagnosis of autoimmune diseases is critical to preventing disease progression.

In their meaningful clinical research study, Ahn et al investigated whether plasma tumor M2-PK is elevated in patients with RA and whether its levels correlate with disease activity. They demonstrated that the level of plasma M2-PK in RA patients is increased, and it is positively correlated with the disease activity. After treatment with immunosuppressive agents the level of M2-PK decreases concomitantly to the reduction of inflammation; however, M2-PK was hardly detected in the synovial tissue.

The differential diagnosis of seronegative RA can be a challenge for clinicians considering the absence of serological biomarkers. In their work, the Italian Group of Bason et al, through the use of random peptide library identified autoantibodies that could be used as serum biomarkers for the diagnosis of this patient population. In particular they found a peptide in the sera of seronegative RA patients and not in healthy controls, that shares homology with some self-antigens, such as Protein-tyrosine kinase 2 beta, B cell scaffold protein, Liprin-alfa1 and Cytotoxic T lymphocyte protein 4.

In the present era, of microarray and high-throughput sequencing analysis, is important to join efforts in comparing and converging the information on gene expression profiles, present in various platforms, to identify potential novel genes and biomarkers.

By selecting five microarray datasets and a high throughput sequencing dataset Zhou et al identified differently expressed genes in immune cell infiltrating the synovial tissues in RA patients as compared to the ones of healthy donors. Authors found that CD8 T cells expressing the alpha chain of the CD8, the chemokine receptor CCL5, the chemokine CXCR4 and Granzyme A are a good diagnostic biomarker candidate for RA. And that CXCR4 activated memory CD4/Tfh cells expressing Granzyme A participate in early pathogenesis of RA. Moreover, RA synovia contained M1 type macrophages, generated by the inflammatory synovial microenvironment, which in turn sustain inflammation and recruitment of monocyte/neutrophils.

Yu et al examined RA’s diagnostic signatures and the effect of immune cell infiltration through integrated bioinformatic analysis and machine-learning strategies, further verified by qRT-PCR.

They found that lymphocyte-specific protein 1 (LSP1), Granulysin (GNLY), and Mesenchymal homobox 2 (MEOX2) regarded as RA’s diagnostic markers and showed their statistically significant difference by qRT-PCR.

Finally, two manuscripts were more focused on RA therapy and predictive biomarkers of therapeutic response.

Hu et al, performed a systematic review on the efficacy of denosumab in patients with RA, focusing on the percent changes in bone mineral density (BMD), and the changes in modified total Sharp score (mTSS), modified Sharp erosion score and joint space narrowing score. Pooled analyses showed that denosumab treatment was superior to bisphosphonates for the improvement of lumbar spine and femoral neck BMD, as well as for the reduction of joint destruction evaluated through mTSS and modified Sharp erosion score.

In their study, Cai et al, have carried out a correlation analysis and single-sample gene set enrichment analysis (ssGSEA) finding 46 common genes between RA patients’ infliximab (IFX)-responders or non-responders and a specific 25-gene signature in datasets of the non-responder patients and Derlin-1 (DERL1) was identified as the hub gene. Interestingly, DERL1 demonstrated to be involved in the immune response and autophagy regulation, since DERL1-siRNA partially inhibited autophagosome formation in RA-fibroblast-like synoviocytes and may have potential predictive value for the therapeutic effect of IFX.

In conclusion, we are pleased that this Research Topic attracted several innovative and valuable scientific articles that faced this issue from different point of view, by using single case reports, real life clinical studies and a plethora of technologies such as bioinformatics and molecular biology.

We hope that this Research Topic will help to share the advances in the knowledge of SARS-CoV-2 infection and vaccination, as well as new potential biomarkers for prompt diagnosis and optimal management of autoimmune rheumatic diseases.

## Author contributions

APD, EN, MML, BL, MMR conceived the Research Topic, revised submitted manuscripts and wrote/revised the Editorial. All authors contributed to the article and approved the submitted version.

## Conflict of interest

The authors declare that the research was conducted in the absence of any commercial or financial relationships that could be construed as a potential conflict of interest.

## Publisher’s note

All claims expressed in this article are solely those of the authors and do not necessarily represent those of their affiliated organizations, or those of the publisher, the editors and the reviewers. Any product that may be evaluated in this article, or claim that may be made by its manufacturer, is not guaranteed or endorsed by the publisher.

